# Healthcare professionals’ experiences of advance care planning conversations with frail older adults admitted to acute geriatric hospital units: a nested qualitative study

**DOI:** 10.1186/s12913-026-15112-x

**Published:** 2026-07-15

**Authors:** Karin Berg Hermansen, Trygve Johannes Lereim Saevareid, Reidar Pedersen, Rigmor Einang Alnes, Linn Brøderud, Maria Romøren, May Helen Midtbust

**Affiliations:** 1 Department of Health Sciences, Faculty of Medicine and Health Sciences, Norwegian University of Science and Technology, Box 1517, Aalesund, 6025 Norway; 2 Centre for Medical Ethics, Institute of Health and Society, University of Oslo, Box 1130 Blindern, Oslo, 0318 Norway; 3 Department of Health and Nursing Science, University of Agder, Agder, Norway; 4 Department of General Practice, Institute of Health and Society, University of Oslo, Box 1130 Blindern, Oslo, 0318 Norway

**Keywords:** Advance care planning, Communication, Older patients, Health personnel, Clinical intervention, Qualitative

## Abstract

**Background:**

Advance care planning can support frail older adults, their next of kin, and healthcare professionals by clarifying patients’ values and preferences and improving communication toward the end of life. Despite these benefits, advance care planning remains underutilized, particularly among frail older adults with complex needs. Limited research has explored healthcare professionals’ experiences with initiating it in acute geriatric settings. As part of a broader research project focused on implementing advance care planning for older adults admitted to acute geriatric hospital units, this study aimed to explore healthcare professionals’ experiences with initiating these conversations.

**Methods:**

A qualitative sub-study, nested within a cluster randomized controlled trial, was conducted with implementation teams and other healthcare professionals from the six geriatric units in the intervention arm. They had recently started engaging in advance care planning with frail, acutely ill older patients and their next of kin. Data were collected through four focus groups and four dyadic interviews, involving a total of 24 participants. The data was analysed by using reflexive thematic analysis.

**Results:**

Four themes developed as perceived benefits: (1) Amplifies the patients’ voice, (2) Improves communication with next of kin, (3) Influences treatment decisions and may avoid overtreatment (4) Contributes to meaningful work. Two themes developed as perceived challenges: (1) Uncertainty regarding the ACP documentation, (2) ACP conversations require dedicated time and focus.

**Conclusion:**

This study revealed that advance care planning conversations in acute geriatric hospital units can amplify patients’ voices and promote their participation in decision-making. Discussions about existential topics appear beneficial and rewarding for patients, next of kin and healthcare professionals, both for in-moment and future decisions. Time constraints may have led to missed opportunities to initiate ACP conversations with patients before the end of life, while documentation issues may have limited their impact on continuity of care.

**Trial registration:**

https://ClinicalTrials.gov Identifier NCT05681585. Registered 03.01.23.

**Supplementary Information:**

The online version contains supplementary material available at https://doi.org/10.1186/s12913-026-15112-x.

## Background

Advance care planning (ACP) is a voluntary communication process that helps people express their preferences and values about future health care [1]. These conversations may contribute to the patient’s wishes being known during challenging decisions toward the end of life, especially if the cognitive ability is expected to be reduced [2, 3]. Multimorbidity increases with age and often leads to a reduced quality of life [4, 5]. Frail, ill older adults are frequently admitted to hospitals, particularly in the final stages of life [6]. In these settings, physicians provide medications to treat and sustain life, and they often face difficult decisions regarding life-sustaining treatment [7]. At the same time, patients and their next of kin are often poorly involved in treatment and care decisions [8, 9] and communication between service levels is often deficient [10, 11]. A consequence of this can either be overtreatment or undertreatment, which leads to significant and unnecessary burdens for patients, next of kin [12] and healthcare professionals (HCPs) [13].

Growing evidence suggests that ACP can address many of these challenges. Participation in ACP has been associated with improved communication quality and reduced decisional conflict [14, 15]. Training in ACP has been shown to improve HCPs competence and confidence in end of life discussions [16]. Systematic reviews indicate that ACP can help HCPs gain deeper insight into what matters most to patients, supporting them in addressing uncertainties and delivering the most appropriate care [17] and may reduce moral distress [18]. ACP may additionally assist HCPs and next of kin to better understand and accept the patient’s wishes toward the end of life [19]. At the same time, there is a growing emphasis on supported rather than substituted decision-making, advocating for greater involvement of patients and next of kin [20], including within the context of ACP [3]. Reviews further indicate that ACP may improve the final phase of life by increasing the right to self-determination, preventing overtreatment and unnecessary hospitalisation [21, 22].

Despite the availability of evidence-based ACP guidelines [1, 3, 23], and the recent publication of national recommendations in Norway [24], a significant gap remains between policies and practices. Its implementation may be hindered by a lack of time, communication skills, documentation routines, reluctance to discuss existential issues, paternalism, and fragmentation [25, 26]. The timing of ACP initiation remains debated, and evidence is still limited regarding context and setting. Research has indicated that ACP is valuable in nursing homes; however, it should be initiated earlier due to many patients having too severe cognitive impairment [27]. These findings underscore the importance of introducing ACP earlier in the trajectory of frailty. ACP conversation with frail older adults is limited [28, 29], and remains underutilized across healthcare services [30, 31]. In hospitals, ACP is only implemented to a small extent, and there is a paucity of knowledge about stakeholders’ experiences in this setting [22, 32].

The present study is nested within a larger project aimed at implementing ACP for frail, home-dwelling older adults admitted to acute geriatric hospital units, and to better include their next of kin, for better participation and co-determination for all the stakeholders [33]. HCPs in acute geriatric units occupy an essential role in identifying appropriate moments to initiate ACP, helping patients reflect on their values, and facilitating communication with next of kin. To our knowledge, research within the Norwegian context is limited, particularly regarding ACP for frail older adults with varying levels of cognitive functioning, and on how to involve next of kin in the process. To address this gap, the present study explored the experiences of HCPs in acute geriatric hospital units as they initiated the first ACP conversation. The study aimed to address the following research question: How did healthcare professionals experience initiating ACP conversations in terms of perceived benefits and disadvantages for patients, next of kin and clinicians?

## Methods

### Study design, intervention and context

This qualitative sub-study was nested within a cluster randomized controlled trial on the implementation of ACP in the routine care for acutely admitted patients in geriatric units [33]. A qualitative exploratory design was used to gain in-depth insight into HCPs experiences with the intervention.

Twelve geriatric hospital units from South-Eastern Norway Health Authority were randomized to either intervention arm, (receiving implementation support) or control arm, (care as usual). The ACP interventions included implementation strategies and the clinical intervention ACP conversations [33]. The chosen strategy for the implementation intervention included an implementation team at each unit. Each team consisted of a coordinator (a nurse or a physician), 1–4 nurses or/and physicians from the unit, with a special interest in ACP. These resource personnel received training and education first, before expanding to all HCPs in the participating units. In addition to structured staff training, managerial endorsement, and a documentation template, an ACP conversation guide (Box 1 and Additional file 1) was developed by the Centre for Medical Ethics (CME). The guide was informed by extensive research and collaboration with healthcare services, patients, and next of kin [34, 35]. It also draws on the Serious Illness Conversation Guide (SICG) [36], and incorporates a two-step model in which patients’ preferences for information and decision-making are assessed first [37]. The questions in the ACP conversation guide served as guidelines and should be adapted to the patient and the situation.

**Box 1:** Advance care planning conversation guide for hospital clinicians

Steps	Questions and content in the advance care planning conversation
1. Give a good start	Briefly explain the purpose of the conversation. Start openly ─ how are you?
2. Information and involvement?	Can you tell…
	• What do you think about your health?
	• What do you know about your health condition, prognosis and healthcare now and in the future?
	• What and how much do you want to know?
	• How much do you want to participate in important healthcare decisions?
	• How much do you want your next of kin to know and be involved?
	Inform about the role of next of kin if the patient cannot consent to healthcare. Provide tailored information about health condition, prognosis and healthcare now and in the future.
3. Values, hopes,	Can you tell...
concerns, and	• What has been and is important in your life?
possibility	• What do you hope for and what is most important to you going forward?
towards the end of	• What worries you?
	• Any thoughts or preferences towards the end of life?
4. Preferences for	What are your thoughts on treatment and other healthcare in the time to come?
future	Is there any treatment or other healthcare you want or do not want in the period to come?
healthcare?	
5. Summarize	Have we understood what is important to you and your wishes for the future?

The intervention involved offering ACP conversation to frail older adults admitted to acute geriatric units and preferably their next of kin. Geriatric beds are primarily allocated for acutely ill, home- dwelling older patients. These patients benefit from a multidisciplinary geriatric team that evaluates their functional and cognitive status, assesses their frailty and creates a personalized plan to maintain their abilities while addressing the acute condition. In Norway the responsible clinician assesses the patient’s capacity to consent and ability to participate in ACP, and to adjust the involvement of the next of kin according to the patient’s capacity and preferences.

Most of the conversations referred to in this data were with home-dwelling patients, > 70 years and Clinical Frailty Scale ≥ 4 [38], and competent to consent to research participation, due to inclusion criteria for the sub-studies during the trial. Before recruitment, guidance on how to inform eligible study participants and how to assess capacity to consent were provided. During the implementation period, physicians led the conversations, reflecting their responsibility for medical decision-making and treatment planning, and ensuring integration of ACP into clinical practice. Nurses played a key role in preparing patients and providing support throughout and following the conversations.

### Participants and recruitment

All participants were employed as HCPs in the various units. Coordinators from each implementation team assisted with providing contact information and organising the interview schedule. KBH and LB contacted participants by email, through which they received written information about the study and a consent form. We gathered data from interviews with all the HCPs in the implementation teams and other eligible HCPs in the six intervention units. We aimed to include nurses and physicians who had participated in ACP conversations. The participants had taken part in between one and ten ACP conversations, with an average of four. Participant characteristics are detailed in Table 1.

**Table 1:** Participants characteristics

	*n* = 24	%
**Age range**		
25–34 35–44 45–54 55–64	7 10 4 3	29.2 41.6 16.7 12.5
**Sex**		
Female Male	18 6	75 25
**Occupation**		
Junior doctor Specialist in internal medicine Specialist in internal medicine + geriatrics Other Nurse	4 3 7 1 9	16.7 12.5 29.2 4.1 37.5
**Years of clinical experience**		
1–3 year 4–9 year 10–15 year > 15 year	4 6 6 8	16.7 25 25 33.3
**Years in attending unit**		
< 1 year 1–3 year 4–9 year 10–15 year > 15 year	3 6 13 1 1	12.5 25 54.2 4.1 4.1
**Role**		
Implementation team Other healthcare professionals	12 12	50 50

### Data collection

Focus groups were used to explore participants’ perceptions and experiences through interactive dialogue [39]. In addition, some interviews were conducted as dyadic interviews, involving two participants interviewed together. This approach was not primarily methodological but reflected the sampling strategy: in some units, only two HCPs met the inclusion criteria, having conducted ACP conversations with patients. In these cases, interviewing them together was considered the most appropriate approach, as dyadic interviews may offer deeper and more nuanced insights than individual interviews alone [40]. All data, whether generated from focus groups or dyadic interviews, were treated as part of the same dataset. A semi-structured interview guide was developed to address the research question, based on previous research at CME [41, 42], and was used in both the focus groups and the dyadic interviews. Following the first two data collection sessions, the authors conducted an initial assessment of the interview guide to ensure alignment with the study aim; no revisions were required. The main questions are presented in Table 2.

**Table 2:** Interview guide

**Questions for the Interviews**
We would appreciate hearing about both the benefits and disadvantages you have experienced with advance care planning. **Experiences with and the significance of advance care planning conversations** • Can you describe advance care planning here at your unit? How common it is, who participates, which patients are not suitable, timing and duration) • What is the significance of these conversations (benefits/disadvantages)? - for patients
- next of kin - healthcare professionals - services • What were the most important themes that emerged during the conversations? (Desires for information/involvement? Wishes for life-prolonging treatment? ) • Is there anything that could have been different in the conversation? (Preparation, patient involvement, type and amount of information) • What role did next of kin play in the conversations? What do you think is the role of next of kin in advance care planning?

The focus groups and dyadic interviews were conducted between December 2023 and October 2024. Four researchers from the implementation study’s project group worked in pairs to co-facilitate each data collection session. KBH (ICU nurse, PhD Fellow) participated in six sessions, LB (RN, PhD Fellow) in six, and TJLS (RN, Assoc. Prof.) and MR (MD and Assoc. Prof.) in two each. In two units, implementation teams and other HCPs participated in separate focus groups to avoid large groups. Four focus groups (3–5 participants) and four dyadic interviews were conducted, involving a total of 24 participants. An overview is provided in Additional file 2. The data collection sessions were conducted in meeting rooms at each participating hospital unit and lasted an average of 85 min, as the implementation process was also explored. All focus groups and dyadic interviews were audio-recorded and transferred to the University of Oslo’s Service for Sensitive Data (TSD), where they were transcribed verbatim.

### Analysis

The analysis was inspired by Braun and Clarke’s six-phase reflexive thematic analysis [43]. Field notes, written after each interview, enriched the analysis by capturing reflections and emotional responses. The first author, KBH, thoroughly listened to all audio recordings and read the transcripts multiple times. Each interview was coded line by line by KBH to capture single meanings in a data-driven orientation, focusing on the overt meanings of data. At the same time, subjectivity informed the process, as the researchers’ perspectives, backgrounds, and ongoing reflections shaped how the data were interpreted and meaning was constructed. Coding and interpretations were discussed between KBH and MHM throughout the process to support reflexive engagement with the data. NVivo 14.0 software [44] was utilized for coding and organizing the dataset. The resulting codes were then structured into initial themes. While the research question provided overall direction, it did not determine the coding process or thematic structure. The analysis was conducted inductively, with coding carried out independently of the research question to allow themes to be developed from patterns of shared meaning in the data. In the later stages of analysis, the themes were considered in relation to the study aim. This approach is consistent with reflexive thematic analysis, in which research questions guide but do not constrain the analytic process [43]. Preliminary themes were discussed collaboratively with TJLS, MHM, and REA, and subsequently shared with all authors. The themes were refined by moving back and forth between data, codes, and themes. All authors engaged in discussions about the preliminary findings and contributed to refining the themes throughout the analytical process.

### Reflexivity

As researchers, KBH, LB, TJLS, RP, and MR, were directly involved in providing implementation support. Our clinical backgrounds and familiarity with the project offered valuable insights but also positioned us as embedded and non-neutral actors in the research process. We recognize that these dual roles may have influenced both how we interpreted participants’ accounts and how they chose to share their experiences. To address this, we actively sought to elicit critical perspectives during interviews and analysis, aiming to present a nuanced and comprehensive account of HCPs experiences.

### Ethical considerations

The study followed the Declaration of Helsinki and was approved by the Norwegian Agency for Shared Services in Education and Research (SIKT), (805491). Written informed consent was obtained from all participants. Participation in ACP conversation was voluntary, with patients being invited and given the option to decline.

## Results

Four themes developed regarding HCPs perceived benefits of ACP: (1) Amplifies the patient’s voice, (2) Improves communication with next of kin, (3) Influences treatmentdecisions and may avoid overtreatment (4) Contributes to meaningful work. Regarding perceived challenges, two themes developed: (1) Uncertainty regarding the ACP documentation, (2) ACP conversations require dedicated time and focus.

### Perceived benefits of ACP

#### Amplifies the patient’s voice

HCPs found that ACP conversation amplified the patient’s voice for them, making it clearer and more impactful. It was new to all HCPs to ask patients how much they wanted to know about their illness and tailoring the information to their preferences. Some reported becoming more attuned to this after ACP intervention, and familiarity with ACP and its tools enhanced their ability to notice hints during the morning medical rounds. By involving the patients, HCPs could focus even more on what the patient wanted and considered important.

*There is a lot of sorrow associated with getting old and frail*,* which perhaps isn’t discussed much. However*,* it’s good because ACP allows them to express it. It’s a sense of loss*,* it’s painful. They have been so incredibly capable and resourceful and then suddenly they need help from others (Nurse*,* FG8.3).*

HCPs told they were accustomed to a holistic approach in geriatric units, addressing both the medical and existential needs of the patients. However, by inviting them to ACP conversation, dedicating time and creating a space for listening, they discovered that the patients remembered it as more personal than usual care. A physician said, *‘The gratitude from the patient and their family highlights how important and eagerly anticipated this is’* (DI6.1). Another said that ACP conversation served as a counteract to the patient’s feeling of being treated as just another case.

*Some patients actually say that they feel like a package [in the hospital]. This [ACP] is a small attempt to give an antidote to that feeling. They get an opportunity to be seen and have the space to express their thought (Physician*,* DI3.2).*

This illustrates how ACP conversations enhanced their understanding of patients’ emotional needs and ensured that patients had the opportunity to express concerns that might otherwise have remained unaddressed.

#### Improves communication with next of kin

HCPs experienced that most patients wanted their next of kin to be present during the ACP conversation. Hence, they found it beneficial when the next of kin could hear what it meant to live well and what mattered the most to the patients. However, they sometimes experienced differing opinions regarding further treatment.

*Sometimes the patient is more realistic because they are old*,* feel tired and worn out. While the next of kin can push*,* be more insistent*,* right? I’ve found it very positive when the next of kin*,* usually children*,* or sometimes spouses*,* get to hear what the elderly actually want (Physician*,* FG2.1).*

Some HCPs noted that, in the units in the general, the next of kin could be drivers of overtreatment.

*In my experience*,* if there is a disagreement*,* it is often because the next of kin wants more than the patient. It goes very well as long as you take the time to explain and talk to them (Physician*,* FG6.5).*

By sharing information during ACP conversations about the patient’s situation and prognosis, HCPs often reached an agreement or provided a more realistic perspective. Some felt they initiated a process, while a nurse found it rewarding to advocate for frail patients.

*They [older patients] feel like they have us [HCPs] supporting them; they are not alone against*,* perhaps three children who want them to continue at any cost. That we are on their team (Nurse*,* FG2.4).*

A physician shared that they had received support from the next of kin during an ACP conversation when a severely ill, old patient had unrealistic expectations about the treatment. They reached an agreement that further treatment was not appropriate due to undesirable side effects. HCPs reported that they facilitated discussions on existential topics between the patient and next of kin, which had never been addressed before.

*They had never talked about death*,* about ‘what we do when I [husband] get worse.’ And he [patient] was really ill at that time. He had been quite ill for a long time. But they had never sat down and had that conversation. So*,* his wife was very pleased that we had*,* in a way*,* opened up the possibility to talk about this (Physician*,* FG6.1).*

Some experienced that several patients had thought a lot about death yet hadn’t put it into words. Other benefits included the next of kin being prepared and their ability to act as the patient’s voice if necessary.

*I think it’s good for everyone involved*,* that it’s clarified with the next of kin. Because if we’ve had an ACP conversation and talked through things that some might not usually discuss*,* then when the unexpected happens*,* or you’re in the middle of a crisis*,* you’re a bit more prepared for what your loved one [patient] wants (Nurse*,* DI5.2).*

This reflection illustrates how ACP conversations may strengthen communication and help prepare next of kin for the patient’s wishes before critical situations arise. This, in turn, may enable HCPs to align care more closely with those wishes.

#### Influences treatment decisions and may avoid overtreatment

One of the most significant changes among HCPs was the recognition of ACP as a tool to clarify the patient’s voice regarding further tests and treatment. They had all experienced overtreatment, difficulties in assessing treatment levels and the next of kin often wanted more. Several physicians were surprised to discover that involving patients often revealed a preference for less intervention and a lower treatment level than initially anticipated.

*We are mostly focused on reaching a decision and on what we*,* as HCPs*,* should do. We haven’t been as concerned with the patient’s own thoughts … We shouldn’t be so afraid to hear what they [patients] really think (Physician*,* DI1.1).*

Some HCPs mentioned that there was no standard practice to ask frail, severely ill older patients about how many rounds of treatment they wanted to undergo once they started, such as pleural tapping or blood transfusions. A nurse said, in the unit generally and before ACP was introduced, *‘I don’t think the frail older patients knew that they had a voice*’ (FG8.4). HCPs recognized the ACP’s benefits when examinations, such as gastroscopy and colonoscopy, were cancelled and chemotherapy was not initiated per the wishes of the patients.

*There is a significant focus on medications and discharge plans*,* but not as much on patients’ understanding of their condition and their perspectives. Do they want this treatment? It’s incredibly interesting to consider. It’s surprising how a half-hour conversation can uncover insights that might save both the patient and the healthcare system from unnecessary issues (Physician*,* DI3.1).*

Some experienced that ACP conversations were different from usual care.

*But there’s something else here [in ACP]*,* because there will be a brief pause where we make sure to ask this question… Yes*,* it is to avoid unwanted overtreatment and troublesome tests (Physician*,* FG2.1).*

HCPs emphasized that documenting patients’ wishes after ACP conversations was valuable. These could assist also less experienced physicians in limiting treatments and in planning the course of action.

*Treatment limitations present significant challenges in today’s healthcare system. There are numerous drivers for conducting investigations and finding minor deviations often compels further examination … This approach alleviates the pressure of feeling responsible for missing a cancer diagnosis or similar conditions. Therefore*,* I believe ACP is highly beneficial to avoid overdiagnosis and overtreatment*,* which are prevalent issues (Physician*,* DI7.1).*

HCPs discussed the ACP’s impact on the utilization of healthcare services. A crucial aspect was the social responsibility of avoiding the excessive use of public resources and preventing what both they and patients consider to be overtreatment. Regarding treatment limitation, a physician said, *‘I don’t do it to save money*,* I do it to prevent suffering’* (DI3.1). Additionally, they believed that this approach could help relocate resources to those who truly need them.

#### Contributes to meaningful work

HCPs experienced that ACP conversations brought meaning and purpose to their workdays. It gave opportunities for reflection, often overlooked in the clinic. Creating space for involvement was considered rewarding.

*I feel that the patients benefit from us creating space for self-determination. I find that to be one of the most rewarding aspects. We set aside time. It’s like pressing a pause button on a hectic day (Nurse*,* FG2.4).*

They felt they had performed a thorough and meaningful job for the patients, contributing something truly beneficial to their care. Some said that it was a good feeling when they saw that it eased patients’ burden a bit after talking about what matters most.

*I definitely think that we are motivated by the positive feedback we receive from patients and their next of kin. We experience that they find it genuinely meaningful*,* and we think it is beneficial for us as caregivers in the long run (Physician*,* FG6.5).*

Positive feedback from patients and next of kin was motivating and all HCPs experienced them to be grateful and satisfied.

*I feel that it has been beneficial for me as well. In such conversations*,* you give a part of yourself and receive something in return … Those are probably the best days when I go home as a physician*,* I think*,* when I’ve had such meaningful conversations (Physician*,* DI3.1).*

HCPs described their emotional investment in patient care as not draining but rather restorative, particularly when interactions were experienced as meaningful. They further reported that patients expressed wishes supported clinical decision-making by guiding physicians in management and could also streamline care during subsequent admissions.

*Many [patients] are quite accepting that they are in the final stage of life. This*,* in a way*,* makes our job a bit easier. It’s not such a sensitive topic*,* really. Rather*,* they might have missed it earlier. They are happy that someone opens that door*,* finally*,* someone who addresses it. They might have been thinking about it for a long time (Physician*,* FG6.5).*

HCPs found it particularly beneficial to understand patients’ wishes before they became too ill or unable to communicate. Some experienced that patients were readmitted in an unconscious state just a few weeks after the ACP conversation. In these cases, HCPs described how documented wishes and prepared next of kin supported emergency department physicians in determining appropriate treatment levels. They perceived that this contributed to the provision of palliative care aligned with patients’ preferences. HCPs described these situations as meaningful, as they felt they facilitated a peaceful end of life.

### Perceived challenges of ACP

#### Uncertainty regarding the ACP documentation

Several HCPs expressed a concern about whether the patient’s wishes were effectively communicated both during readmissions and within healthcare services, to the general practitioner and nursing home.

*It was just by chance that the junior doctor in the emergency department saw that an ACP conversation had been conducted*,* noted it in the previous discharge summary and planned accordingly. But really*,* it was almost lucky*,* given how the system is at present.*

*Several next of kin have told me that it [ACP conversation] needs to go further in the system. They say it should reach the general practitioner*,* the nursing home*,* and the health centre doctor. And I completely agree*,* but I don’t know if we can manage it (Physician*,* FG2.1).*

They reported receiving crucial information about the patients’ future priorities and felt responsible for sharing it with others involved. This may indicate an underlying concern among HCPs that the patient’s wishes were at risk of being overlooked.

Another challenge highlighted was that, due to other clinical duties, the notes from the ACP conversation were not always distributed for the patient and next of kin to review.

*It is crucial to have a solid foundation*,* ensuring that they agree with what is written about them. In the worst-case scenario*,* such a situation could lead to a breach of trust (Physician*,* DI3.1).*

This may have hindered HCPs from gaining reassurance that they had accurately understood the patient’s wishes. At the same time, many found it reassuring to provide a verbal summary at the end of the conversation to ensure alignment with the patient’s intentions. Furthermore, all participants had been informed that their expressed wishes could be revised, which might have contributed to a shared understanding of the ACP conversation as an ongoing process.

#### ACP conversations require dedicated time and focus

HCPs found it challenging to integrate ACP conversations into daily clinical routines due to time constraints and the need to prioritize patient assessment and treatment. Managing ACP conversations, especially when dealing with severely ill patients in the unit, was challenging. They emphasized the importance of having the time to listen. Some of them turned off notifications to stay focused; however, they found it uncomfortable. A physician called it ‘*the art of staying calm when stressed’* (DI1.1).

They frequently described that they found it time-consuming to organize ACP conversations and difficult to coordinate the schedules of all parties involved, including nurses, physicians and next of kin. They noted that while there were several eligible patients, limited capacity dictated who was prioritized and invited.

*What influences who we choose [to invite] is partly capacity*,* because there are probably several we could have chosen*,* but there’s no time*,* as there are too many patients we need to visit. So*,* we know this is something we could have done for more (Nurse*,* FG8.1).*

HCPs experienced that openly discussing patients’ hopes, worries and wishes could be time-consuming as they were often unsure of what might come up. This made the duration unpredictable. Some reported skipping lunch and working overtime to complete ACP.

*I don’t find it that difficult*,* but I might be more concerned that if I have that conversation*,* I’ll have to work an extra hour of overtime to finish everything. That’s the issue … Because you never know*,* if you set aside time for such a conversation*,* it can take longer than expected*,* making it unpredictable how much time you’ll have left for other tasks (Physician*,* DI5.1).*

HCPs did not find ACP conversations themselves challenging, but the required time had made them hesitant to initiate them. This may have resulted in missed opportunities to amplify patients’ voices.

## Discussion

This study provides novel knowledge of HCPs experiences with ACP conversations in acute geriatric hospital units. Communication with patients and their next of kin through ACP enhances patient participation. From HCPs perspective, discussions on existential topics prove to be beneficial and rewarding for patients, next of kin and HCPs, both for immediate and future decisions.

Our study found that ACP conversations amplified patients’ voices and influenced treatment decisions based on their preferences. Physicians were surprised that some frail, older inpatients wanted less treatment than suggested or expected. This highlights a potential mismatch between patient priorities and clinical assumptions, where a focus on treatment and cure may make it challenging to consider withholding treatment. Strict adherence to guidelines can lead to unnecessary tests and treatments. According to Kherad et al., creating a shared decision-making process between patients and physicians serves as a key mechanism for preventing this [45]. Our findings extend this by illustrating how ACP may operationalize shared decision-making in practice, thereby supporting patient involvement.

Our findings underscored that involving next of kin significantly improved communication, particularly regarding patients’ values, preferences and decision-making, and enabled them to represent the patient’s voice when needed. This appeared to occur through clarifying patients’ values, ensuring continuity of information, and supporting shared understanding, thereby aligning expectations and reducing uncertainty in decision-making. This aligns with another sub-study [46], where next of kin emphasized the importance of HCPs in facilitating discussions towards end of life, preparing them to actively contribute to collective decision-making. The promotion of patient autonomy and participation through ACP is further supported by studies highlighting the value of triadic agreements and shared information [47], which have been associated with improved relationships and care tailored to patients’ preferences [48]. Taken together, these findings suggest that improved communication in ACP is not only about information exchange, but about creating a shared understanding among patients, next of kin, and HCPs.

Additionally, our findings indicated that ACP conversations positively influenced HCPs personal satisfaction, particularly by helping to avoid burdensome tests for patients (i.e., unnecessary or invasive tests with limited clinical benefit) and reducing the risk of overtreatment. While Gomes Souza et al. showed that ACP benefited HCPs by helping them understand patients’ goals and values, thereby guiding decision-making [49], the personal satisfaction reported by HCPs in our study appears to be a novel contribution. This suggests that ACP may not only improve person-centred care but also reduce moral stress and support professional motivation, although its potential impact on motivation warrants further investigation.

ACP is not solely about end-of-life or about reducing life-sustaining treatment. However, HCPs found the intervention beneficial as it enabled a step-by-step approach to acknowledging imminent death, guided by the patient’s preferences for discussion topics and what mattered most to them. Discussing death and dying in hospital can be challenging [50]. A study in Norwegian somatic hospital units found that physicians avoided discussing existential topics [51]. However, in this study, HCPs did not experience such conversations as challenging. Instead, they reported that several patients and their next of kin were grateful for this support and found it meaningful. The difference may be attributed to the context, particularly the experiences of HCPs in geriatric units managing end-of-life care. This suggests that barriers to these conversations may be more closely related to clinical practice patterns than to reluctance among patients or HCPs. A Lancet report highlighted that ‘bringing death back into life’ is valuable and needed [52]. Several illnesses faced by patients in geriatric units cannot be fully resolved. By exploring their values, hopes and worries, ACP can be a resource for how patients can be supported to live in the best way towards the end of life. Thus, ACP may facilitate a shift from disease-focused care to person-centred care in contexts with limited curative options.

The present study probed perceived disadvantages in geriatric units when ACP conversations actually took place. HCPs noted no disadvantages, but faced challenges, including documentation issues and time constraints. This indicates that challenges are primarily systemic rather than inherent to ACP itself.

Despite identifying only a few challenges, its usefulness may be limited by the absence of documentation systems that effectively communicate patients’ wishes within the hospital and between different levels of healthcare services. Although such integration is recognised as crucial for coordinated, effective care [3] and appropriate review [1], it remains difficult to achieve in practice. Patients’ real-time preferences may be difficult to share and document across healthcare settings [53, 54], potentially leading to decisions being made without full awareness of patients’ values and preferences. Documentation that conveys ACP conversations with direct relevance for clinical decision-making, such as patients’ values, goals and treatment preferences is therefore essential, yet may be insufficient if systems do not support consistent use. Together, this highlights the need for integrated systems that ensure ACP information is accessible, regularly updated, and clinically actionable. Moreover, our study indicated that outcomes are achieved during the ACP conversation itself, including improved understanding between patients, next of kin and HCPs, and greater preparedness for future decisions. Exploring what matters most to patients may lead to care that is better aligned with their preferences and experiences that are perceived as meaningful. This was in line with McMahan et al., who found that in-moment discussions where patients were actively listened to, and next of kin were prepared through collective decision-making held greater value than documentation alone [55]. Taken together, these findings suggest that ACP should be understood as both a communicative process and a documentation practice, and that focusing on one without the other may limit its impact.

HCPs in this study demonstrated a willingness to invest time in ACP conversations, viewing it as a valuable part of the treatment. However, they found it to be time-consuming, as noted in other contexts by Lovell et al. [56], highlighting the complexity of decision-making in clinical practice [57]. Limited time for patient conversations can hinder thorough discussions about treatment options, leading to non-beneficial treatments [58]. In the present study, patients expressed wishes provided valuable support for decision-making, helping HCPs navigate complex situations and potentially avoid overtreatment. This suggests that ACP may represent a time investment that improves downstream efficiency and decision quality. This may also contribute to time savings during future hospital admissions. However, creating space for ACP conversations remains challenging, highlighting the need to explicitly integrate them as a core component of clinical care, including allocation of dedicated time, integration into routine workflows, and organizational support.

### Strengths and limitations

This study integrates a qualitative component within a cluster randomized controlled study, uniquely positioning it to explore HCPs experiences with the intervention [59]. Conducted across six geriatric units at different hospitals, it captured variation in clinical practice. Through interviews with HCPs actively engaged in ACP conversations, the study provides valuable insight into how ACP can contribute to person centred care in acute geriatric hospital units.

However, several limitations should be acknowledged. The study was limited to intervention units, which may influence the transferability of the findings. In addition, participants had attended only a few structured ACP conversations. Those who dedicated time to the intervention may have been more motivated, potentially increasing the likelihood of reporting positive experiences. Furthermore, the results are based on the patients’ first ACP conversation, and we did not explore the wider or long-term impact of ACP in healthcare services.

## Conclusions

This nested qualitative study demonstrated a range of benefits with ACP conversations. It helped HCPs identify patients’ values and preferences, guiding in-time decisions and serving as a tool for future healthcare. Another key benefit was the prevention of unnecessary tests and overtreatment. However, logistical and documentation challenges were found to be significant, which may reduce the potential impact. This underscores the importance of addressing these challenges, and further research is needed to identify sustainable solutions.

## Supplementary Information

Below is the link to the electronic supplementary material.

Supplementary Material 1: Advance care planning conversation guide for hospital clinicians.

Supplementary Material 2: Overview of focus groups and dyadic interviews.

## Data Availability

The dataset will be de-identified or deleted before the end of the study period, in line with the research ethics approval and consent. If it is possible to de-identify, and subsequently anonymize the data, they will be made publicly available.

## Abbreviations

ACP: Advance care planning
CME: Centre for Medical Ethics
HCPs: Healthcare professionals
SIKT: Norwegian Agency for Shared Services in Education and Research
TSD: The University of Oslo’s Service for Sensitive Data
