# Supplementary material for: Healthcare professionals’ experiences of advance care planning conversations with frail older adults admitted to acute geriatric hospital units: a nested qualitative study
Source: BMC Health Serv Res. 2026 Jul 15;26:1380. doi: 10.1186/s12913-026-15112-x (PMC13659965; doi:10.1186/s12913-026-15112-x)
Supplement: Supplementary file 1 [file 12913_2026_15112_MOESM1_ESM.pdf]

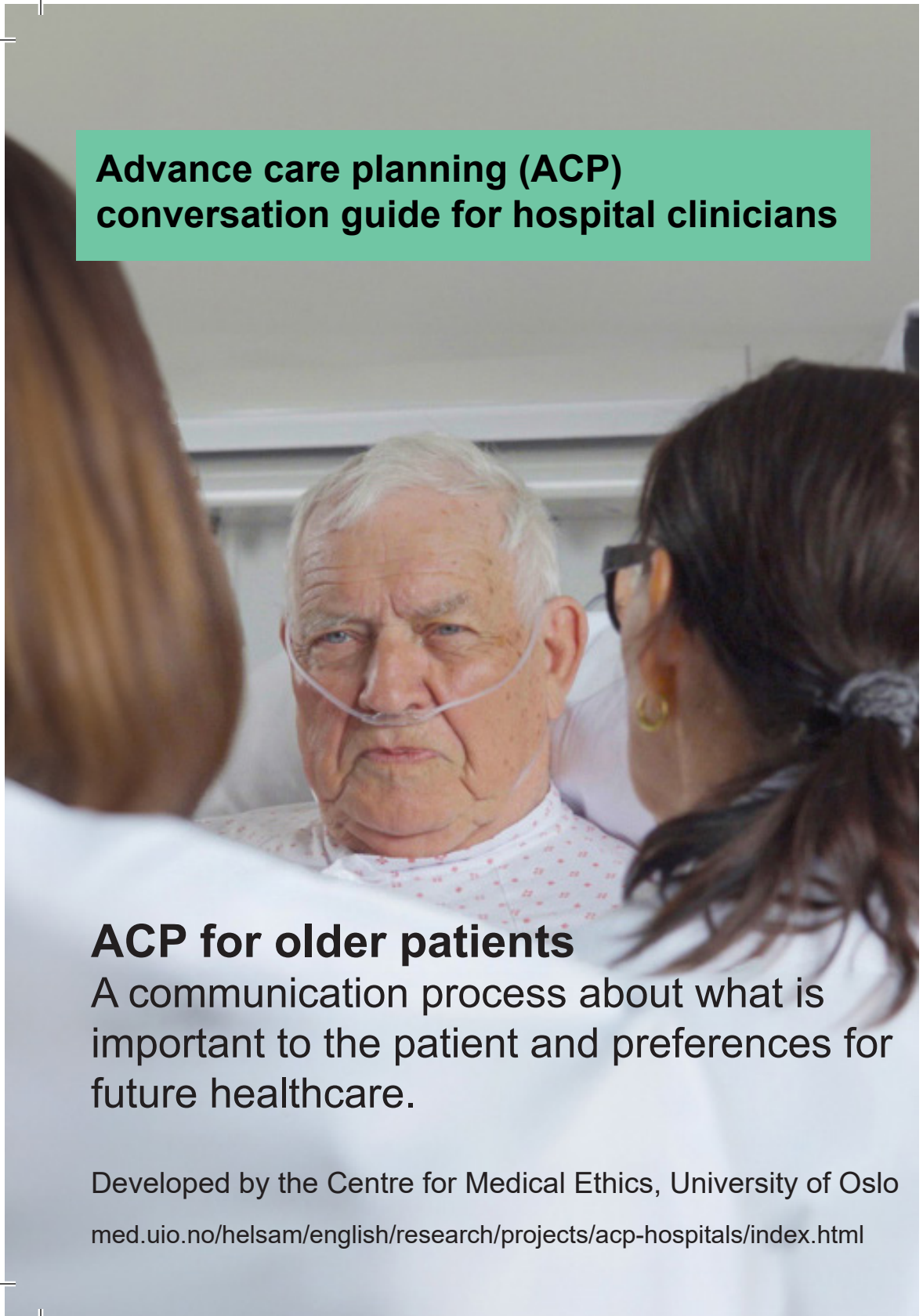

Advance care planning (ACP) conversation guide for hospital clinicians

ACP for older patients

A communication process about what is important to the patient and preferences for future healthcare.

Developed by the Centre for Medical Ethics, University of Oslo

med.uio.no/helsam/english/research/projects/acp-hospitals/index.html

Before Assess the patient's ability to consent to participate. Determine if the next-of-kin should be invited. Extend an invitation ensuring the option to decline.

During Create a good atmosphere. You may use the 'Ask-tell-ask' technique in the communication. The questions in this guide should be adapted to the patient and the situation.

After Document the conversation in the medical record and the discharge summary.

We recommend that the patient, possibly the next-of-kin, is given the opportunity to read through the documentation. It should be informed that:

- The content is shared with the general practitioner and others who will provide important healthcare to the patient, so that the conversation can continue there.
- These professionals will be encouraged to invite the patient, and possibly next-of-kin, to a new conversation later.

Steps	Questions and content in the advance care planning conversation
**1. Give a good start**	Briefly explain the purpose of the conversation. Start openly — how are you?
**2. Information and involvement?**	Can you tell…<br>• What do you think about your health?<br>• What do you know about your health condition, prognosis and healthcare now and in the future?<br>• What and how much do you want to know?<br>• How much do you want to participate in important healthcare decisions?<br>• How much do you want your next-of-kin to know and be involved?<br><br>Inform about the role of next-of-kin if the patient cannot consent to healthcare. Provide tailored information about health condition, prognosis and healthcare now and in the future.
**3. Values, hopes, concerns, and possibility towards the end of life?**	Can you tell...<br>• What has been and is important in your life?<br>• What do you hope for and what is most important to you going forward?<br>• What worries you?<br>• Any thoughts or preferences towards the end of life?
**4. Preferences for future healthcare?**	What are your thoughts on treatment and other healthcare in the time to come?<br>Is there any treatment or other healthcare you want or do not want in the period to come?
**5. Summarize**	Have we understood what is important to you and your wishes for the future?
