# Supplementary material for: Healthcare professionals’ experiences of advance care planning conversations with frail older adults admitted to acute geriatric hospital units: a nested qualitative study
Source: BMC Health Serv Res. 2026 Jul 15;26:1380. doi: 10.1186/s12913-026-15112-x (PMC13659965; doi:10.1186/s12913-026-15112-x)
Supplement: Supplementary file 2 [file 12913_2026_15112_MOESM2_ESM.pdf]

Additional file 2. Overview of focus groups and dyadic interviews

To preserve participant anonymity, interviews were assigned anonymised numeric identifiers (1–8); FG denotes focus groups and DI denotes dyadic interviews.

Table S1. Overview of focus groups and dyadic interviews

Data collection method	Group/Interview ID	Hospital	Participants (n)	Profession
Focus group	FG2	B	4	3 nurses, 1 physician
Focus group	FG4	D	3	1 nurse, 2 physicians
Focus group	FG6	C	5	5 physicians
Focus group	FG8	B	4	4 nurses
Dyadic interview	DI1	A	2	1 nurse, 1 physician
Dyadic interview	DI3	C	2	2 physicians
Dyadic interview	DI5	E	2	1 nurse, 1 physician
Dyadic interview	DI7	F	2	2 physicians
**Total (n)**			**24**	
